# The Physicochemical, Nutritional, and Antioxidative Properties of Plant‐Based Quinoa Yogurt

**DOI:** 10.1002/fsn3.70910

**Published:** 2025-09-10

**Authors:** Qi Wang, Qianyu Li, Hong Zhang, Jingtong Wu, Wenjun Qin, Jin Cai

**Affiliations:** ^1^ School of Life Science Shanxi University Taiyuan Shanxi China; ^2^ Shanxi Wutai Mountain Tianyu Agricultural Development Co., Ltd. Taiyuan Shanxi China; ^3^ Materials and Chemical Engineering Taiyuan University Taiyuan Shanxi China; ^4^ Nutritional Department Shanxi Traditional Chinese Medical Hospital Taiyuan Shanxi China; ^5^ Institute of Applied Chemistry Shanxi University Taiyuan Shanxi China

**Keywords:** antioxidation, nutritional peculiarity, physicochemical characteristic, plant‐based yogurt, quinoa, simulated digestion

## Abstract

In previous work, a kind of pure plant‐based quinoa yogurt was obtained through *Streptococcus salivarius* subsp. *thermophilus* and *Lactobacillus delbrueckii* subsp. *bulgaricus* fermentation. This study evaluated its physicochemical, nutritional, and antioxidative properties. The results showed the sensory score of quinoa yogurt was 88.33 ± 1.21 points. The contents of protein, fat, and total sugar were 3.60% ± 0.01%, 2.08% ± 0.02%, and 17.76 ± 1.17 mg/g, respectively, making it a high‐protein, low‐sugar, and low‐fat beverage. The essential amino acid and total amino acid contents of quinoa yogurt were 5.61 ± 0.21 mg/g and 15.56 ± 0.69 mg/g (*p* < 0.05), respectively. A series of organic acids such as lactic acid and succinic acid were produced. Forty‐one types of volatile flavor compounds were detected; among them, the content of 2‐pentylfuran was the highest (55.84 ± 20.97 mg/100 g), granting the yogurt a unique bean aroma, fruit aroma, and vegetable aroma. The combined action of these flavor substances enhanced the acceptance and nutritional value of the yogurt and further influenced color, texture, and storage stability. Additionally, the total phenols and DPPH radical scavenging ability of the quinoa yogurt were significantly increased (*p* < 0.05), endowing the yogurt with antioxidant properties. Moreover, the simulated digestion significantly increased the total phenols and antioxidant capacity of the yogurt. These results demonstrated that quinoa yogurt is a very good health drink.

## Graphical abstract

The nutritional value of quinoa yogurt has been enhanced. The types and contents of organic acids and volatile flavor substances in the quinoa yogurt have been enhanced, and they were higher than those in commercial yogurt. Simulating the digestive process can enhance the total phenolic content and antioxidant activity of quinoa yogurt.

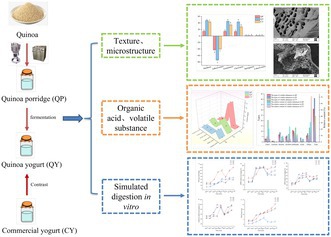

## 1 Introduction

Plant‐based food refers to food made from plants or their products as raw materials, which can be processed through certain techniques with or without the addition of other plant ingredients. It has characteristics similar to animal food in terms of shape, flavor, and texture, but does not contain animal components (Li et al. 2024). Regular intake of these substances can help prevent and treat diseases such as diabetes and cancer (Zhai et al. 2025). At present, plant‐based foods were mainly divided into five categories: plant‐based meat products, plant‐based dairy products, plant‐based egg products, plant‐based frozen products, and other plant‐based foods (Chen and Luo 2024).

Plant‐based yogurt refers to a beverage made from various plants through fermentation. According to data from Old Republic International (ORI), the comprehensive market growth rate of global plant‐based yogurt was as high as 17.8% from 2018 to 2023, and it is expected to reach 6.46 billion US dollars in 2027 (Zannini et al. 2018), presenting a broad market prospect.

*Chenopodium quinoa* Willd. is a gluten‐free dicotyledonous false grain, which is the only type of complete‐nutrition grain recognized by the Food and Agriculture Organization of the United Nations (FAO) (Liu et al. 2023). It is rich in protein (15% ~ 19%), essential amino acids (EAA), vitamins, minerals, and dietary fiber (6% ~ 21%) (Manzanilla‐Valdez et al. 2024). In addition, quinoa is a good source of bioactive components such as polyphenols and flavonoids, and has antibacterial, antioxidant, anti‐inflammatory, and blood pressure‐lowering properties (Guo et al. 2025).

Fermentation can enhance the digestibility and bioavailability of nutrients, generate or release bioactive substances (Rodríguez et al. 2021), and also reduce the content of antinutrient substances (Sharma et al. 2025).

At present, most of the quinoa yogurt was prepared by adding quinoa to milk to obtain compound milk (Abd‐Rabou et al. 2020; Codină et al. 2016), which cannot be regarded as true plant‐based quinoa yogurt. In our previous work, the lactic acid bacteria (LAB) was used to ferment the variety “Tai Li No. 2” to obtain a pure plant‐based quinoa yogurt. In this paper, the nutritional and antioxidant characteristics of quinoa yogurt were explored. This work provided a good choice for gluten allergies, lactose intolerances and vegetarians.

## 2 Materials and Methods

### 2.1 Materials

Quinoa was sourced from Tianyu Agricultural Co. Ltd. in Wutai Mountain, Shanxi Province, specifically the variety of “Tai Li No. 2” (The contents of protein, fat and carbohydrate were 16.9%, 6.0%, and 61.0%, respectively). The *Streptococcus salivarius* subsp. *thermophilus* and *Lactobacillus delbrueckii* subsp. *bulgaricus* were purchased from DANISCO (Aarhus, Denmark).

### 2.2 Preparation of Quinoa Yogurt

In our previous work, the quinoa porridge (QP) was the material for quinoa yogurt (QY), and the optimal fermentation process was established as follows: fermentation time of 8.3 h, fermentation temperature of 43.4°C, inoculation amount of 1.2% (w/v), and white sugar addition of 4.2% (w/v). Under these conditions, pure plant‐based QY was produced and subsequent experiments were conducted. A commercial yogurt (CY) was used as a control.

### 2.3 Determination of Physicochemical Indicators and Microbial Number

#### 2.3.1 The Sensory Scores and Chroma

The yogurt samples were evaluated by sensory methods as previously described (He et al. 2024).

The sensory evaluation panel consisted of food science students (10 males and 10 females) who had received professional sensory training. The yogurt samples were dispensed into standardized tasting cups for assessment. During the evaluation, participants rated various sensory attributes, including color, texture, flavor, taste, and assigned scores accordingly. The experimental protocol was reviewed and approved by the Ethics Review Committee for Scientific Research at Shanxi University (Approval No. SXULL2025119). All participants were fully briefed on the procedures and provided voluntary consent to participate in the study. Personal and sensory data collected from participants were treated as strictly confidential and were used exclusively for research purposes. Written informed consent was obtained from all participants regarding the collection and use of their personal and experimental data.

The chroma of the samples was analyzed by using a colorimeter (Jeske et al. 2017). The results were expressed by L* (luminance), a* (red to green), b* (yellow to blue) and WI (white index), and the instrument was calibrated with standard black and white plates.

#### 2.3.2 The pH, Titration Acidity, Water Holding Capacity and Viscosity

The pH value was directly measured by a pH meter. The titration method of potentiometry was used to determine the titration acidity (TTA) according to the following procedures: the ultrapure water was boiled and cooled to room temperature, followed by mixing with the samples at a ratio of 2∶1 (v/v). The mixture was titrated with NaOH standard solution, and the titration was complete when the pH meter reached 8.3. The water holding capacity (WHC) was respectively determined according to the methods described by Ma et al. (2025). A certain mass of samples was weighed into a centrifuge tube and was centrifuged at 4°C at 4000 rpm for 30 min, the supernatant was discarded, and the total weight of the remaining sample and the centrifuge tube was recorded, the calculation of WHC was as follows:

$$\mathrm{WHC}\;\left(\%\right)=\frac{m_{3}-m_{1}}{m_{2}-m_{1}}\times 100$$

Where m_1_ represented the weight of the centrifuge tube (g); m_2_ represented the weight of the sample (g); m_3_ represented the sum of the weight of the sample after centrifugation and the centrifuge tube (g).

The viscosity of the samples was measured by a viscometer with a No. 4 rotor and a rotational speed of 60 rpm.

#### 2.3.3 The Texture and Microstructure

The texture of QP, QY, and CY was analyzed by TA touch Texture Analyzer according to the method by Júnior et al. (2025). A 35 mm cylindrical probe was adopted; the probe speed before, during, and after measurement was 5 mm/s, 1 mm/s, and 1 mm/s, respectively. The target displacement was 15 mm; the time interval between two probe tests was 5 s. The hardness, adhesiveness, springiness, cohesiveness, gumminess, and chewiness of the samples were analyzed.

The microstructure was observed following the method by Kamal‐Eldina et al. (2020) with certain modifications. A small amount of freeze‐dried QP and QY samples was taken out, gold‐plated, and scanned in all areas. They were operated at a voltage of 3 KV to obtain microscopic images at magnifications of 200×, 350×, and 800×, respectively. The contrasted images were extracted and observed.

#### 2.3.4 The Number of Lactic Acid Bacteria

The number of viable LAB (The *Streptococcus salivarius* subsp. *thermophilus* and *Lactobacillus delbrueckii* subsp. *bulgaricus*) of yogurt was determined by traditional spread plate count at 37°C.

### 2.4 Evaluation of Nutritional and Antioxidative Properties

#### 2.4.1 The Basic Nutritional Components

The protein and fat contents were determined according to the method by Oğuz et al. (2022). The total solids content (TSC) was determined by the gravimetric method (Ujiroghene et al. 2019). A certain mass of the samples was weighed and placed in the beaker, dried for 2 h in a drying oven at (100 ± 5)°C, followed by cooling in the desiccator for 0.5 h, then taken out and weighed. Repeat this step until the difference in mass between the two times was no more than 1 mg. The TSC was calculated as follows:

$$\mathrm{TSC}\;\left(\%\right)=\frac{m_{1}-m_{2}}{m_{1}-m_{3}}\times 100$$

Where m_1_ represented the total mass of the beaker and sample (g); m_2_ represented the total mass of the beaker and sample after drying to constant weight (g); m_3_ represented the mass of the beaker after drying to constant weight (g).

The total sugar content was determined according to the method by Zavřel et al. (2018). 0.5 mL of phenol and 2.5 mL of concentrated sulfuric acid were added to 2 mL of the samples solution. After being mixed well, it was placed at room temperature and left to stand for 20 min. It was measured for its absorbance value at a wavelength of 490 nm and calculated the content based on the glucose standard curve, and the result was expressed in mg/g. The reducing sugar content was determined by Poe and Edson (1932). 1.5 mL of 3, 5‐dinitrosalicylic acid reagent (DNS) and 1 mL of ultra‐pure water were added to 1 mL of the samples solution. The mixture was heated in a boiling water bath for 5 min and then cooled to room temperature, followed by diluting with distilled water to 20 mL. Then, the absorbance was measured at 540 nm. The reducing sugar content was calculated based on the glucose standard curve, and the result was expressed in mg/g. Then, the sugar components in the samples were further determined by high performance liquid chromatography (LC).

#### 2.4.2 The Amino Acids

Following the method of Walsh and Brown (2000), amino acid composition and content of QP, QY, and CY were determined by an automatic amino acid analyzer.

#### 2.4.3 The Organic Acids

The organic acid contents of QP, QY, and CY were determined according to the method by Zhai et al. (2024). The samples were centrifuged, and the supernatants were filtered through a 0.45 μm water phase filter membrane, and the filtrate was eluted with a C18 column at 40°C with a 10 μL injection volume and detected at 210 nm. The results were expressed in mg/g.

#### 2.4.4 The Volatile Flavor Compounds

The volatile flavor substances were determined according to the method by Martínez‐Onandi et al. (2017). Five gram of QP, QY, and CY were weighed respectively and placed in a headspace vial. Then, 5 mL of ultrapure water, 2 g of NaCl, and 15 μL of 2‐octanol (0.041 mg/mL, w/v) were added. The top glass vials were placed in a water bath at 45°C for equilibrium for 30 min. The temperature ramping procedures were as follows: maintain at 50°C for 5 min, increase at 5°C/min to 120°C, increase at 10°C/min to 230°C, and maintain for 5 min; the injection port temperature was 250°C, and the samples were injected without split.

#### 2.4.5 The Total Phenolic Content and Antioxidant Activity

##### 2.4.5.1 Preparation of the Samples Extract Solution

The samples extract solution was prepared according to the method of Trigueros et al. (2014). The samples were precisely weighed and placed into a 50 mL centrifuge tube, 15 mL of acidified methanol (containing 0.3% HCl, v/v) was added, and subjected to shear extraction at 10,000 rpm for 5 min. Then, they were placed (−20°C, 1 h) to ensure that proteins were completely precipitated; afterward, the samples were centrifuged at 4°C (7000 rpm, 15 min), the supernatant was collected and diluted to 50 mL. These supernatants were used as sample test solutions and stored for future use.

##### 2.4.5.2 Determination of the Total Phenolic Content

The total phenolic content (TPC) was determined according to the method of Anuyahong et al. (2020). Specifically, the sample test solution was mixed evenly with the Folin‐Phenol reagent in a ratio of 1:5 (v/v), and the reaction was carried out for 5 min; subsequently, 3 mL of Na_2_CO_3_ solution (7.5%, w/v) was added, and the solution was incubated in the dark for 1 h; the absorbance was then measured at a wavelength of 765 nm. A standard curve was constructed using gallic acid, and the TPC was calculated according to the standard curve.

##### 2.4.5.3 Determination of the Antioxidant Capacity

The 2,2‐diphenyl‐1‐picrylhydrazyl (DPPH) scavenging abilities of QP, QY, and CY were determined referring to the method of Lee et al. (2018) with slight modifications. Sixty milligram of ascorbic acid was weighed and diluted to 1 L in a volumetric flask to obtain the standard solution mother liquid (60 mg/L, w/v); the mother liquid was diluted with distilled water to prepare different concentrations of ascorbic acid solutions; the sample test solution was mixed with the DPPH working solution (0.2 mol/L, w/v) in a ratio of 1∶1 (v/v) and reacted in the dark for 30 min, and the control group and blank control were set with absolute ethanol and ultrapure water, respectively. The absorbance values were measured at a wavelength of 517 nm; the calculation formula was as follows:

$$\text{DPPH scavenging ability}\;\left(\%\right)=\frac{A_{1}-\left(A_{2}-A_{3}\right)}{A_{1}}\times 100$$

Where A_1_ represented the absorbance value measured by using anhydrous ethanol as the substitute sample solution; A_2_ represented the absorbance value of the sample solution to be tested; A_3_ represented the absorbance value measured by using anhydrous ethanol as the substitute DPPH solution.

The 2, 2′‐azino‐bis‐(3‐ethylbenzothiazoline‐6‐sulfonic) acid (ABTS) scavenging abilities of QP, QY, and CY were determined according to the slightly modified method of Paşayeva et al. (2025). Ten milligram of ascorbic acid was diluted to 1 L volumetric flask to obtain the standard solution mother liquid (10 mg/L, w/v); the mother liquid was diluted with distilled water to prepare different concentrations of ascorbic acid solutions. The sample test solution and the ABTS working solution were mixed in a ratio of 1∶2 (v/v) and then reacted in the dark for 10 min, and the control group was set with absolute ethanol. The absorbance values were measured at a wavelength of 734 nm; the calculation formula was as follows:

$$\text{ABTS scavenging ability}\;\left(\%\right)=\left(1-\frac{A_{1}}{A_{0}}\right)\times 100$$

Where A_0_ represented the absorbance value measured using anhydrous ethanol to replace the substitute samples solution, A_1_ represented the absorbance value of the samples solution to be tested.

The Hydroxyl radical scavenging abilities of QP, QY, and CY were determined referred to the method of Hwang et al. (2021) with minor modifications. Ten milligram of ascorbic acid was diluted to 50 mL volumetric flask to obtain the standard solution mother liquid (200 μg/L, w/v); the mother liquid was diluted with distilled water to prepare different concentrations of ascorbic acid solutions. 1 mL of each solution and the sample test solution was taken; 1 mL of FeSO_4_ solution (9 mmol/L, w/v), salicylic acid solution (6 mmol/L, w/v) and H_2_O_2_ solution (20 mmol/L, v/v) were added, and were placed in a water bath at 37°C for 20 min. The absorbance values were measured at a wavelength of 510 nm; the calculation formula was as follows:

$$\text{Hydroxyl radical scavenging activity}\;\left(\%\right)=\left[1-\left(\frac{A_{2}-A_{1}}{A_{0}}\right)\right]\times 100$$

Where A_0_ represented the absorbance value measured using ultra‐pure water to replace the substitute samples solution; A_1_ represented the absorbance value measured using ultra‐pure water to replace the H_2_O_2_ solution; A_2_ represented the absorbance value of the samples solution to be tested.

The ferrous reducing antioxidant power (FRAP) of QP, QY, and CY was determined using the slightly modified method of Anuyahong et al. (2020). Ten milligram of ascorbic acid was diluted to 1 L in a volumetric flask to obtain the standard solution mother liquid (10 mg/L, w/v); the mother liquid was diluted with distilled water to prepare different concentrations of ascorbic acid solutions. One milliliter of each solution and the sample test solution was taken, 2.5 mL of PBS buffer solution (0.2 mol/L, pH 6.6) and potassium ferricyanide solution (1%, w/v) were added. After mixing evenly, it was placed in a water bath at 50°C for 20 min, and then 2.5 mL of trichloroacetic acid was added (10%, w/v); afterwards, they were centrifuged at 4000 rpm for 10 min at 4°C. Finally, 2 mL of supernatant was mixed with 0.5 mL of ferric chloride solution (1%, w/v) and 2.5 mL of ultra‐pure water, and the absorbance values were measured at a wavelength of 750 nm.

### 2.5 The Total Phenolic Content and Antioxidant Capacity During Simulated Gastrointestinal Digestion Process In Vitro

#### 2.5.1 Simulation Gastrointestinal Digestion

The digestive fluid was prepared according to the method by Brodkorb et al. (2019). The samples were mixed with the saliva containing amylase, the pH of the digestion solution was adjusted to 7 with NaOH solution, and were digested at 37°C at 200 rpm for 2 min. The digestion solution from the previous stage was mixed with the gastric juice containing pepsin, the pH was adjusted to 2 with HCl solution, and was digested at 37°C at 200 rpm for 2 h. The digestion solution from the previous stage was mixed with the intestinal fluid, the pH was adjusted to 7 with NaOH solution, and was digested at 37°C at 200 rpm for 2 h. The temperature and pH were kept constant during the digestion process.

#### 2.5.2 Determination of the Total Phenolic Content and Antioxidant Capacity

The TPC and antioxidant activity during in vitro simulated digestion of QP, QY, and CY were determined, and the methods were the same as those in 2.4.5.

### 2.6 Quality Changes of Yogurt During Storage

The trends of TTA, pH, WHC, viable bacteria count (The *Streptococcus salivarius* subsp. *thermophilus* and *Lactobacillus delbrueckii* subsp. *bulgaricus*) and sensory score of QY stored at 4°C over a period of 21 days was determined, with tests conducted every 3 days.

### 2.7 Statistical Analysis

All the experiments were conducted in parallel three times. The results were expressed as mean ± standard deviation. A one‐way ANOVA analysis was conducted using IBM SPSS Statistics (version 26.0), and Tukey's test was employed for multiple comparisons. It was considered statistically significant at *p* < 0.05. Graphs were drawn using Origin 2025.

## 3 Results and Discussion

### 3.1 Physicochemical Indicators and Microbiological Analysis

#### 3.1.1 The Sensory Scores and Chroma

**FIGURE 1:**
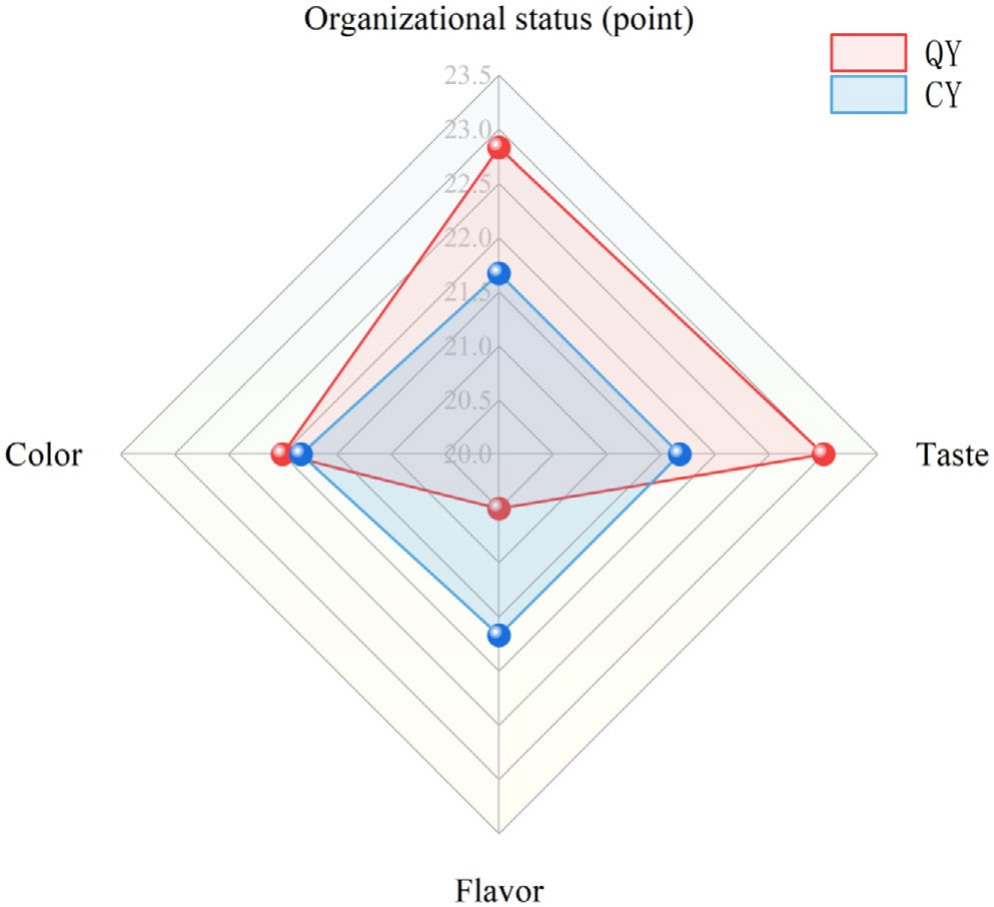
The sensory scores of QY and CY. QY and CY refer to quinoa yogurt and commercial yogurt, respectively.

The sensory flavor profile of QY and CY was shown in Figure 1; it was seen that QY has a harmonious flavor, a uniform texture, no whey precipitation, an appropriate sweet and sour taste, and a color similar to the characteristic light brown of quinoa. The sensory score was slightly higher than that of CY. This might be because the triterpenoid saponin compounds in quinoa were removed during the preparation of QP, the bitterness was reduced (Guo et al. 2024), and the flavor was well‐received.

The chroma of the QP, QY, and CY was shown in Table 1. As can be seen from the table, the L*, a*, b*, and WI values of the QY were significantly higher than those of the QP and the CY (*p* < 0.05). The WI value was relatively high, which may be favored by consumers. This result might be due to the fact that after the QP was fermented, the changes in the protein and fat contents led to an increase in L* of the QY, the red and yellow colors have been intensified, and an increase in WI as well (Teichert et al. 2020). In addition, Jeske et al. (2017) found that the varieties of different raw materials and processing techniques may also cause color changes in yogurt, and the influence was significant.

**TABLE 1:** The chroma of QP, QY and CY.

	L*	a*	b*	WI
QP	63.52 ± 0.08^b^	0.08 ± 0.02^b^	8.02 ± 0.02^b^	62.65 ± 0.08^b^
QY	69.8 ± 1.59^a^	0.24 ± 0.07^a^	8.42 ± 0.20^a^	68.65 ± 1.57^a^
CY	58.47 ± 0.14^c^	−2.22 ± 0.03^c^	0.56 ± 0.06^c^	58.40 ± 0.14^c^

*Note:* Data are expressed as the mean ± standard deviation. Different lowercase letters in the same column indicate significant differences among the samples (*p* < 0.05). L* represented the brightness; a* represented the participation of red (+) and green (−) colors of components; b* represented the participation of yellow (+) and blue (−) colors of components; WI represented the white index. QP, QY, and CY referred to quinoa porridge, quinoa yogurt, and commercial yogurt, respectively.

#### 3.1.2 The pH, Titration Acidity, Water Holding Capacity, Total Solids Content, and Viscosity

In yogurt, the TTA and pH were negatively correlated. As seen from Table 2, the TTA of QY was extremely significantly lower than that of CY (*p* < 0.001). This may be because QP was pure plant‐based and does not contain lactose, and the carbon source utilized by LAB was reduced. These LAB can only use quinoa as the substrate and white granulated sugar as the carbon source for fermentation (Davoodi et al. 2016; Maoloni et al. 2024). There was no significant difference in WHC, TSC, and viscosity content between the two types of yogurt (*p* > 0.05).

**TABLE 2:** The physicochemical composition of QY and CY.

	QY	CY
TTA (^。^T)	38.63 ± 0.45***	71.48 ± 0.63***
pH	4.43 ± 0.01***	3.80 ± 0.01***
WHC (%)	49.93 ± 0.81	47.72 ± 1.38
TSC (%)	17.18 ± 0.63	17.20 ± 0.63
Viscosity (mPa·S)	5029.00 ± 59.10	4989.67 ± 24.19

*Note:* Data are expressed as the mean ± standard deviation. *** indicated significant differences among different samples in the same row (*p* < 0.001). QY and CY referred to quinoa yogurt and commercial yogurt, respectively.

#### 3.1.3 The Texture and Microstructure

The textural analysis of QP, QY, and CY was shown in Table 3. Hardness was the most direct indicator reflecting the taste of the product, which directly affects the adhesiveness, gumminess, and chewiness (Zannini et al. 2018). As seen in Table 3, the hardness, adhesiveness, gumminess, and chewiness of QY were significantly higher than those of QP and CY (*p* < 0.05). Zhao et al. (2021) found that the increase in protein and fat content helps to achieve gel cross‐link inside yogurt, further increasing the hardness and thus resulting in a more solid and dense structure. The springiness and cohesiveness of QP and QY were not significantly changed, but both were significantly higher than those of CY (*p* < 0.05).

**TABLE 3:** The texture of QP, QY and CY.

	Hardness (g)	Adhesiveness (g·sec)	Gumminess	Chewiness	Springiness	Cohesiveness
QP	14.42 ± 0.18^b^	−42.94 ± 3.93^b^	14.11 ± 0.42^c^	13.75 ± 0.85^c^	0.97 ± 0.04^a^	0.98 ± 0.02^a^
QY	47.67 ± 0.66^a^	−74.23 ± 3.63^a^	45.47 ± 2.65^a^	43.59 ± 4.23^a^	0.96 ± 0.04^a^	0.95 ± 0.04^a^
CY	43.60 ± 2.68^c^	−40.18 ± 1.75^c^	34.60 ± 3.24^b^	28.51 ± 2.41^b^	0.82 ± 0.03^b^	0.79 ± 0.03^b^

*Note:* Data are expressed as the mean ± standard deviation. Different lowercase letters in the same column indicate significant differences among the samples (*p* < 0.05). QP, QY, and CY referred to quinoa porridge, quinoa yogurt, and commercial yogurt, respectively.

**FIGURE 2:**
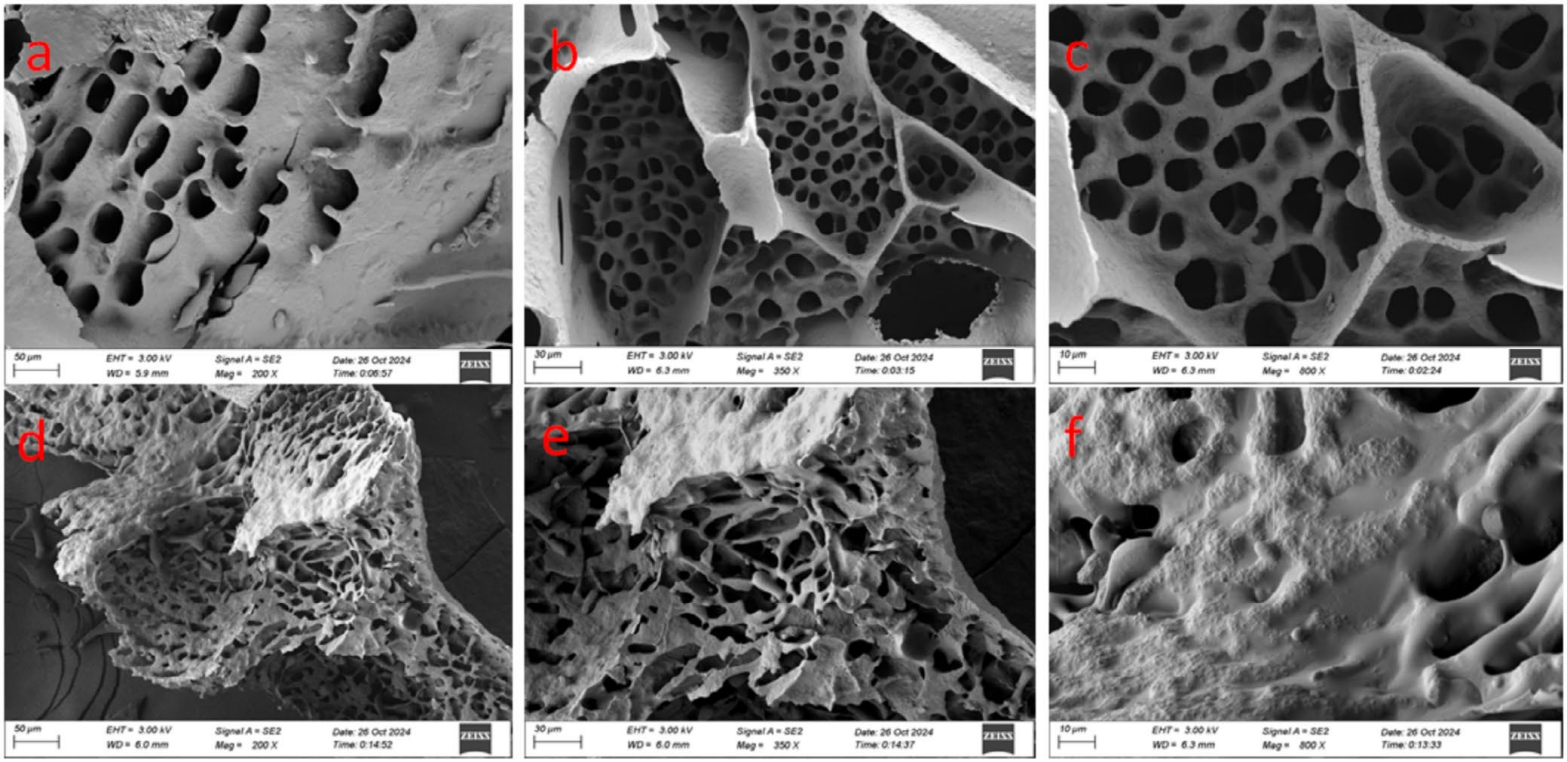
Microscopic structure images of QP and QY: (a–c) referred to images of QP at magnifications of 200×, 350×, and 800×, respectively; (d–f) referred to images of QY at magnifications of 200×, 350×, and 800×, respectively. QP and QY referred to quinoa porridge and quinoa yogurt, respectively.

As shown in Figure 2, the change in textural characteristics may be related to the microstructure. The internal gel structure of the QP was loose and fragmented and filled with irregular pores (Figure 2a–c). After fermentation, the internal structure of QY was more compact, and the pores were significantly reduced; a stable fibrous net‐like three‐dimensional structure was formed (Figure 2d–f). This uniform and dense three‐dimensional network structure was conducive to the attachment of extracellular polysaccharides produced by probiotics (Xu et al. 2018). The extracellular polysaccharide can improve the WHC and viscosity of yogurt (Ren et al. 2025). These further affect the precipitation of whey during storage.

#### 3.1.4 The Number of Viable Bacteria

The number of viable bacteria (The *Streptococcus salivarius* subsp. *thermophilus* and *Lactobacillus delbrueckii* subsp. *bulgaricus*) in the QY was 4.54 × 10^8^ CFU/g, while that of commercial plant‐based yogurt were 10^6^ ~ 10^8^ CFU/g. It was generally recognized that viable bacteria in yogurt have the functions of anticancer, regulating intestinal flora, relieving constipation, and lowering cholesterol (Mathur et al. 2020).

### 3.2 Evaluation of Nutritional and Functional Properties

#### 3.2.1 Analysis of Basic Nutritional Components

As can be seen from Table 4, the protein content of QY was 3.60% ± 0.01%, which was significantly higher than that of CY. The yogurt made from quinoa was rich in pure plant protein, which can replace animal protein and delay unhealthy aging (Ortolá et al. 2019). It also helps maintain the stability of blood sugar in diabetics to a certain extent (Viguiliouk et al. 2015). The fat content of QY was 2.08% ± 0.02%, which was significantly lower than that of CY (*p* < 0.001). Therefore, QY is beneficial to human health.

**TABLE 4:** The nutritional components of QP, QY and CY.

	QP	QY	CY
Protein (%)	2.31 ± 0.01^c^	3.60 ± 0.01^a^	3.20 ± 0.02^b^
Fat (%)	1.13 ± 0.03^c^	2.08 ± 0.02^b^	3.78 ± 0.04^a^
Reducing sugar (mg/g)	17.37 ± 0.86^b^	14.73 ± 0.66^c^	62.98 ± 0.84^a^
Total sugar (mg/g)	23.01 ± 2.32^b^	17.76 ± 1.17^c^	182.20 ± 0.86^a^
Glucose (mg/g)	6.54 ± 1.13^a^	00 ± 00^b^	0.17 ± 0.05^b^
Sucrose (mg/g)	7.76 ± 0.69^b^	7.63 ± 1.51^b^	26.11 ± 4.46^a^
Maltose (mg/g)	0.53 ± 0.03^b^	0.7 ± 0.01^a^	0.18 ± 0.02^c^
Lactose (mg/g)	00 ± 00^b^	00 ± 00^b^	4.15 ± 0.31^a^

*Note:* Data are expressed as the mean ± standard deviation. Different lowercase letters in the same row indicate significant differences among the samples (*p* < 0.05). QP, QY, and CY referred to quinoa porridge, quinoa yogurt, and commercial yogurt, respectively.

The results of the sugar components and contents of QP, QY, and CY were shown in Table 4. The contents of reducing sugar and total sugar in QY were significantly reduced (*p* < 0.05), which were utilized by LAB for acid production and flavor substance generation (Zhou et al. 2025). This result was consistent with the conclusion of Gengatharan et al. (2021). Compared with CY, the total sugar and reducing sugar contents of QY were significantly lower (*p* < 0.05), which gave QY the characteristic of being low in sugar. On this basis, the various sugar components were qualitatively and quantitatively analyzed; the content of sucrose in QY was the highest, followed by fructose and maltose. Compared with CY, QY has a much lower sugar content, which made it an excellent choice for people who have specific requirements for sugar intake.

#### 3.2.2 Analysis of the Amino Acids

**FIGURE 3:**
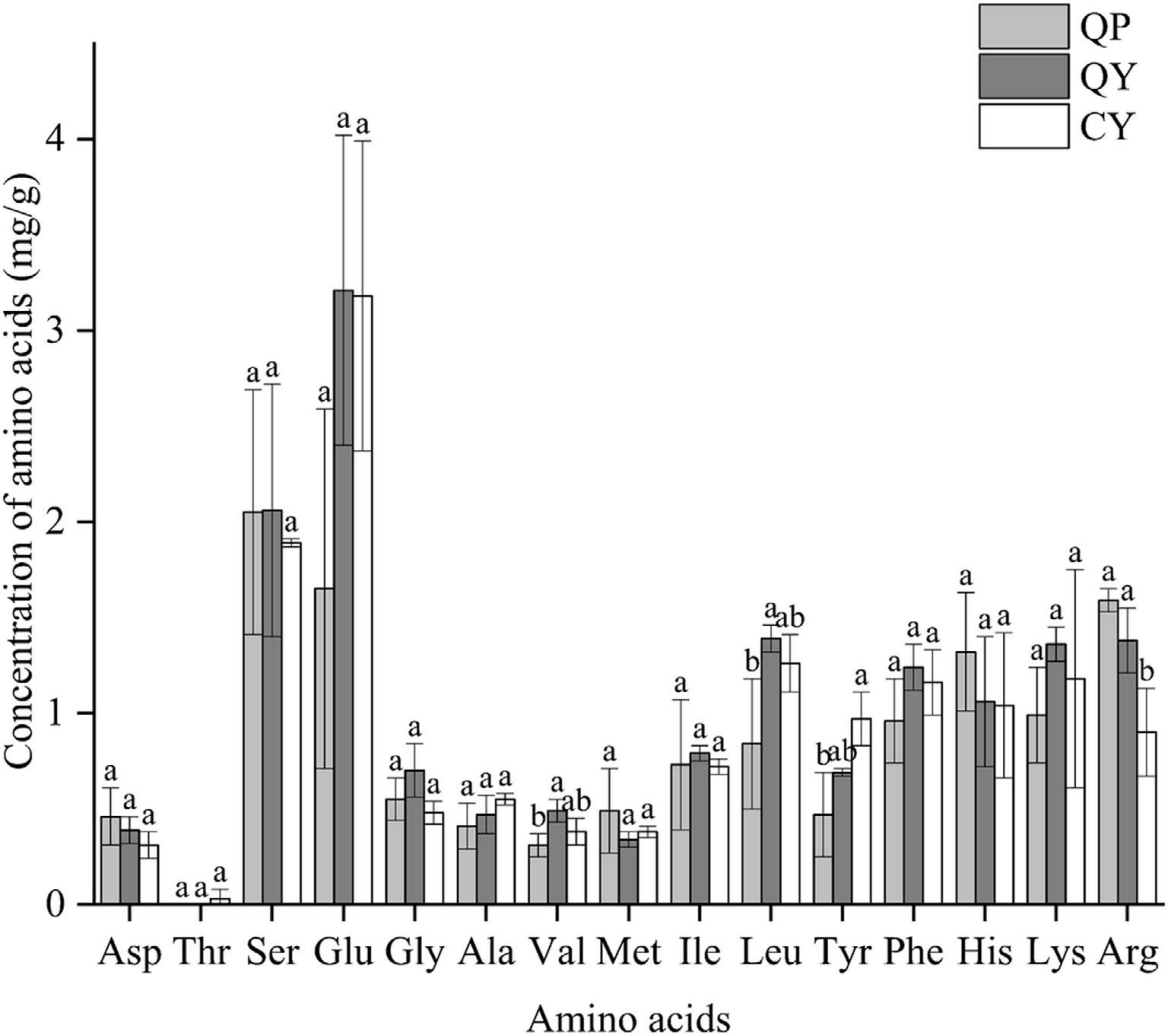
The amino acid composition and content of QP, QY, and CY. Data are expressed as the mean ± standard deviation. Different lowercase letters within the same amino acid indicate significant differences among the samples (*p* < 0.05). QP, QY, and CY refer to quinoa porridge, quinoa yogurt, and commercial yogurt, respectively.

The types and contents of amino acid in QP, QY, and CY were shown in Figure 3. The essential amino acid content and total amino acid content of QY were significantly higher than those of QP (*p* < 0.05), with increases of 29.86% and 21.37%, respectively. Some studies have found that when QP was fermented by LAB, casein and whey protein were converted into smaller molecule peptides and amino acids that are easier to absorb (Azambuja et al. 2017). After the QP has been homogenized, part of the quinoa protein was unfolded, charged amino acids were exposed on the protein surface, the particle size of the protein was reduced, the interaction between the protein and the solvent was promoted (Zhao et al. 2023; Luo et al. 2022), and the solubility was increased.

Among the 16 amino acids detected in QY, Glutamate (Glu) has the highest content, followed by Serine (Ser) and Arginine (Arg). Glu was an important neurotransmitter in cells, participating in the internal energy metabolism activities of the human body, which can alleviate acute and chronic hyperammonemia and other diseases, and improve the brain diseases and liver diseases induced by them, and has a certain detoxification effect on the body (Cauli et al. 2009). Ser was used to help the body form phospholipids needed for producing cells, and also participates in brain development, neuronal excitation, and other activities, and plays a certain role in alleviating schizophrenia (Balu 2016) and antidepression (Malkesman et al. 2012). Arg not only controls the synthesis of proteins in the body, participates in nonspecific immune regulation and antioxidant reactions in the body (Wang et al. 2021), but also participates in physiological functions such as cell metabolism, vasodilation, and synthesis of other amino acids (Patil et al. 2016). The results demonstrated that QY has a higher drinking value.

#### 3.2.3 Analysis of the Organic Acids

As can be seen from Table 5, compared with QP, fermentation increased the content and types of organic acids in QY. These organic acids served as precursor substances for flavor substances and further influenced the flavor, quality, acidity, and consumer sensory perception of yogurt (Ndhlala et al. 2022). Compared with QP, the contents of lactic acid and succinic acid in QY were significantly increased to 4.64 ± 0.83 mg/g and 1.67 ± 0.21 mg/g (*p* < 0.05), respectively, but the content of malic acid was significantly decreased by 25.00%, possibly due to the fact that malic acid was converted into mild lactic acid and succinic acid during fermentation (Sniffen et al. 2006). Moreover, the fermented yogurt had a more harmonious taste and reduced irritating sourness (Sniffen et al. 2006). Among them, lactic acid was produced by LAB through fermentation of Glu and was the most important organic acid in dairy products, which was of great significance for the quality and flavor formation of dairy products (Costa et al. 2016). Succinic acid was an important intermediate metabolite in the tricarboxylic acid cycle and the core for maintaining energy stability and cellular metabolism (Wan et al. 2020). In CY, only lactic acid and citric acid were detected, indicating that its taste was not richer than QY due to the few types of organic acids.

**TABLE 5:** Contents of organic acids in QP, QY and CY.

Types	QP	QY	CY
Tartaric acid (mg/g)	00 ± 00^a^	00 ± 00^a^	00 ± 00^a^
Malic acid (mg/g)	2.76 ± 0.54^a^	2.07 ± 0.08^b^	00 ± 00^c^
Lactic acid (mg/g)	00 ± 00^c^	4.64 ± 0.83^b^	10.93 ± 0.22^a^
Acetic acid (mg/g)	00 ± 00^a^	00 ± 00^a^	00 ± 00^a^
Citric acid (mg/g)	00 ± 00^b^	00 ± 00^b^	0.36 ± 0.05^a^
Succinic acid (mg/g)	00 ± 00^b^	1.67 ± 0.21^a^	00 ± 00^b^

*Note:* Data are expressed as the mean ± standard deviation. Different lowercase letters in the same row indicate significant differences among the samples (*p* < 0.05). QP, QY, and CY referred to quinoa porridge, quinoa yogurt, and commercial yogurt, respectively.

#### 3.2.4 Analysis of the Volatile Flavor Compounds

**FIGURE 4:**
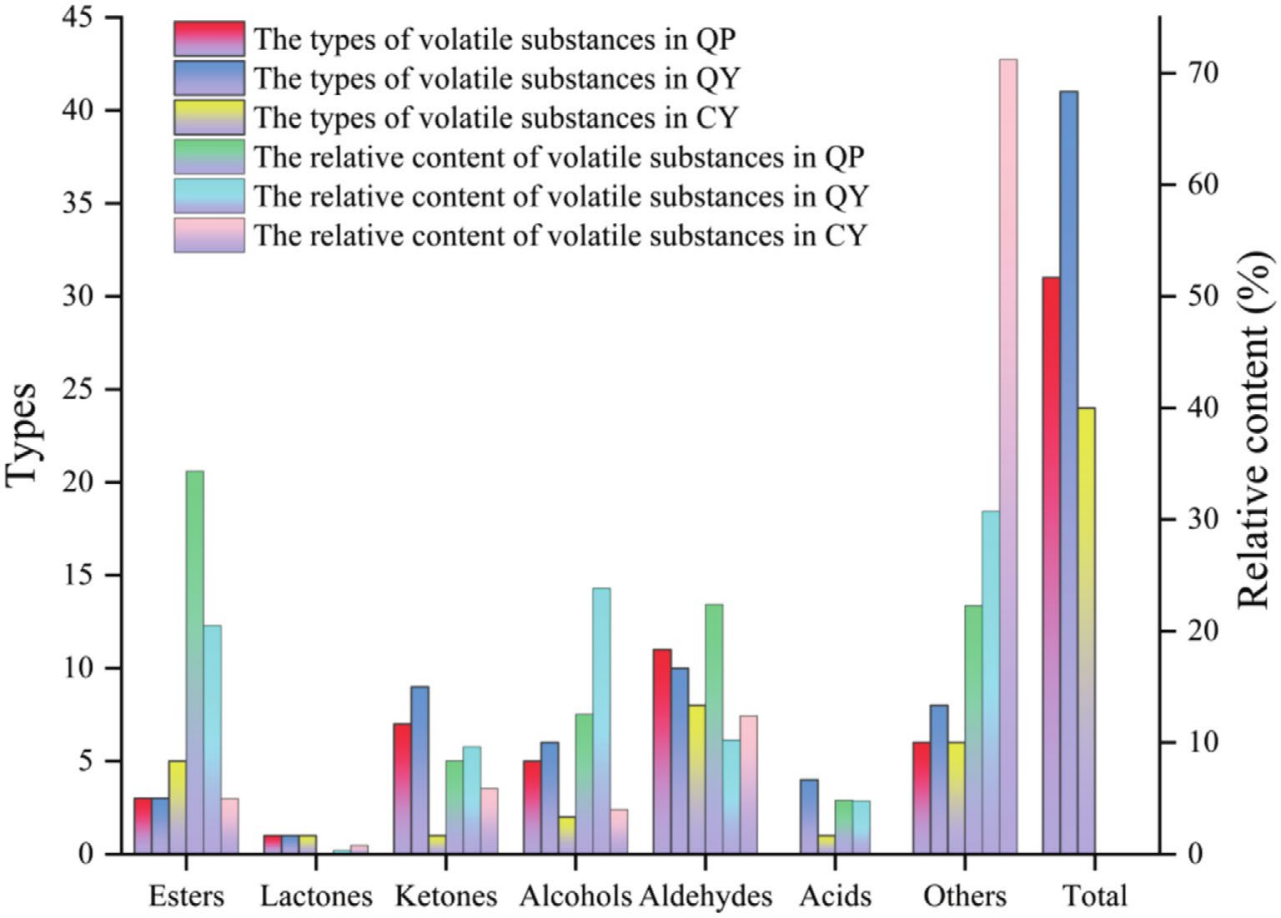
The types and relative contents of volatile substances of QP, QY, and CY. QP, QY, and CY refer to quinoa porridge, quinoa yogurt, and commercial yogurt, respectively.

The protein, fat, sugar, and other compounds contained in QP were partially preserved during fermentation by LAB, while the other part undergoes Maillard reaction, glycoside hydrolysis, fatty acid oxidation, and other reactions by LAB to produce esters, acids, aldehydes, ketones, alcohols, and other flavor compounds (Fan et al. 2022). The unique aroma of QY was caused by the combined action of these flavor compounds. As shown in Figure 4 and Table 6, a total of 41 volatile compounds were detected in QY, including 3 types of esters, 1 type of lactone, 9 types of ketones, 6 types of alcohols, 10 types of aldehydes, 4 types of acids, and 8 types of other compounds. From Figure 4, the highest relative content of QY was accounted for by other compounds (30.72%), followed by alcohols (23.83%), esters (20.48%), aldehyde (10.21%) and ketones (9.60%), respectively. Alcoholic substances can be converted into ester substances under the action of ethanol acetyltransferase, endowing yogurt with a distinctive fruity aroma (An et al. 2023). In Table 6, the most compound in QY was 2‐pentylfuran, reaching 55.84 ± 20.97 mg/100 g. In previous reports, the furan compounds were produced from the Maillard reaction influenced by the different types of amino acids and reducing sugars in the raw materials, which endowed yogurt with unique bean, fruit, and vegetable aromas (Li et al. 2025). Among esters and alcohols, the two compounds with the highest content in QY were butyl acetate (44.47 ± 15.79 mg/100 g) and 2‐propyl‐1‐heptanol (34.47 ± 4.68 mg/100 mg), respectively. Aldehyde compounds were the most diverse type of flavor compounds detected in QY, with 5‐hydroxymethylfurfural exhibiting the highest content (11.73 ± 3.88 mg/100 g). Peleteiro et al. (2015) studied that furfural compounds were produced by the Maillard reaction or caramelization reaction, and not only contributed to the flavor of yogurt, but also exhibited good biological effects, such as antioxidant activity and inhibition of red blood cell pathology (Li et al. 2011). Compared to these substances, there were fewer types and contents of acidic substances, which may be because some acidic substances were converted into ester substances (An et al. 2023) under the action of corresponding ethanol acetyltransferases and esterases.

**TABLE 6:** The types and contents of volatilized flavor substances in QP, QY and CY.

	Types	Aroma content/(mg/100 g)			Present the flavor
		QP	QY	CY	
**Esters**					
1	Ethyl pyruvate	36.20 ± 3.50	ND	ND	Floral and fruity aroma
2	Butyl acetate	43.17 ± 2.91	44.47 ± 15.79	ND	Fruit fragrance
4	Methyl oleate	0.50 ± 0.02	ND	ND	Lipid flavor
5	2‐Methylpentyl formate	ND	16.22 ± 3.79	ND	
6	Nonyl chloroformate	ND	0.53 ± 0.24	ND	
7	Ethyl caproate	ND	ND	3.91 ± 0.73	Sweetness and fruity flavor
8	Butyl butyryllactate	ND	ND	7.48 ± 3.76	Soft and creamy fragrance
9	Ethyl caproate	ND	ND	1.11 ± 0.62	Coconut fragrance
10	Methyl chloroacetate	ND	ND	1.80 ± 0.91	Pungent odor
11	Ethyl octanoate	ND	ND	1.77 ± 0.94	Pineapple and apple fragrance, brandy aroma
	A total of 11	79.87	61.22	16.07	
**Lactones**					
12	γ‐Octalactone	0.17 ± 0.09	ND	2.54 ± 2.83	The aroma of coconuts
13	4‐Dodecanolide	ND	1.02 ± 0.24	ND	Cream, peach, and pear aroma
	A total of 2	0.17	1.02	2.54	
**Ketones**					
14	2‐Hexanone	13.73 ± 9.18	4.06 ± 1.58	ND	Fruity, spicy, herbal fragrance
15	2 (5H)‐Furanone	0.88 ± 0.78	0.35 ± 0.18	ND	Butter flavor
16	4‐Methyl‐2‐heptanone	0.52 ± 0.42	ND	ND	Scent of cinnamon
17	3‐Methylcyclopentan‐1,2‐dione	0.22 ± 0.09	ND	ND	Caramel fragrance, nutty aroma
18	1‐(3‐Butyl‐2‐epoxyethenyl)ethanone	1.09 ± 0.09	1.06 ± 0.30	ND	
19	5‐Methyl‐2‐hexanone	ND	5.91 ± 3.58	ND	A pleasant smell
20	3,4‐Epoxycyclopentanone	ND	0.53 ± 0.36	ND	Mint flavor
21	2‐Nonanone	ND	1.68 ± 0.67	18.96 ± 6.29	Fragrance of magnolia, rose and tea
22	2‐Methyl‐3‐hydroxy‐4‐pyrone	ND	0.96 ± 0.09	ND	Toasted and sweet cream flavor
23	2‐Methyl‐4‐octanone	2.44 ± 1.94	ND	ND	
24	2‐Dodecanone	0.58 ± 0.12	ND	ND	Fruit aroma
25	2‐Undecanone	ND	7.18 ± 4.41	ND	Fruit flavor
26	2‐Tridecanone	ND	6.95 ± 3.09	ND	Wax fragrance, coconut fragrance, nut fragrance and herbal fragrance
	A total of 13	19.46	28.68	18.96	
**Alcohols**					
27	2‐Phenylethanol	ND	ND	8.65 ± 3.52	Rose fragrance
28	2‐Ethylhexanol	ND	ND	4.21 ± 1.02	Sweetness, floral fragrance
29	Cis‐1,2‐cyclohexanediol	0.40 ± 0.25	ND	ND	
30	1‐Nonanol	0.26 ± 0.08	1.12 ± 0.18	ND	Rose fragrance
31	1‐Hexadecanol	1.14 ± 0.89	ND	ND	Rose fragrance
32	Furfuryl alcohol	1.61 ± 0.82	0.49 ± 0.14	ND	A distinctive pungent and spicy odor
33	n‐Hexyl alcohol	25.77 ± 0.87	29.86 ± 10.37	ND	Fruity aroma, wine aroma
34	2‐Propyl‐1‐heptanol	ND	34.47 ± 4.68	ND	
35	1‐Octanol	ND	2.58 ± 1.01	ND	The flavor of the wine
36	2‐Hexyl‐1‐decanol	ND	2.69 ± 1.03	ND	Special alcohol and fat flavors
	A total of 10	29.18	71.21	12.86	
**Aldehydes**					
37	Hexanal	25.79 ± 0.87	ND	ND	Fruit fragrance
38	3‐Furaldehyde	0.52 ± 0.21	1.9 ± 0.27	ND	
39	Heptaldehyde	5.03 ± 0.68	0.79 ± 0.54	ND	Fruit fragrance
40	(E)‐2‐hepten‐1‐aldehyde	1.00 ± 0.29	1.78 ± 0.76	ND	Pungent odor
41	Benzaldehyde	10.61 ± 0.42	3.15 ± 1.88	3.66 ± 1.01	The smell of bitter almonds
42	(E,E)‐2,4‐Hexadienal	0.23 ± 0.13	ND	ND	Fatty aldehyde odor
43	Nonanal	3.84 ± 0.67	2.04 ± 2.00	8.39 ± 0.25	Mint and wax floral fragrance
44	Isoamylaldehyde	0.38 ± 0.22	ND	ND	Apple fragrance
45	Decanal	0.56 ± 0.22	0.38 ± 0.07	2.47 ± 0.53	Sweet orange and lemon scents, along with an aroma of oil.
46	5‐Hydroxymethylfurfural	3.89 ± 2.01	11.73 ± 3.88	4.98 ± 1.20	Spring yellow chrysanthemum fragrance
47	Lauryl aldehyde	0.26 ± 0.12	0.38 ± 0.13	1.32 ± 1.11	Violet, woody scent
48	2,2‐Dimethyl‐3‐hydroxypropionaldehyde	ND	7.83 ± 1.63	ND	Pungent odor
49	Trans‐2‐nonenal	ND	0.54 ± 0.20	ND	The smell of cucumbers and grass
50	2‐Methylpentanal	ND	ND	ND	The aroma of fried peanuts
51	2,5‐Dihydroxybenzaldehyde	ND	ND	16.27 ± 7.91	
52	Undecanal	ND	ND	1.72 ± 0.91	Rose fragrance
53	Tetradecanal‌‌	ND	ND	1.24 ± 0.62	The scents of oil, iris, and peach
	A total of 17	52.11	30.52	40.05	
**Acids**					
54	Isovaleric acid	ND	1.48 ± 0.67	ND	Sweet and fruity aroma
55	2‐Methylbutyric acid	ND	0.42 ± 0.26	ND	The smell of goat cheese
56	Hexanoic acid	ND	7.71 ± 2.49	ND	The smell of coconut oil
57	2‐Ethyl‐4‐methylpentanoic acid	ND	4.73 ± 4.10	ND	Fruit aroma, strawberry fragrance
58	Octanoic acid	ND	ND	15.39 ± 5.92	Weak fruity aroma
	A total of 5	0	14.34	15.39	
**Others**					
59	2,3,3‐Trimethylpentane	4.47 ± 2.33	4.90 ± 0.73	ND	
60	2,4‐Dimethylheptane	41.09 ± 13.07	ND	68.63 ± 8.70	Special odor
61	4‐Methylheptane	2.3 ± 0.43	ND	38.19 ± 5.89	
62	Tridecane	0.38 ± 0.14	ND	ND	Special hydrocarbon odor
63	3,3,4‐Trimethylhexane	1.81 ± 0.41	ND	ND	
64	2,4‐Dimethylhexane	ND	16.24 ± 3.49	ND	
65	5‐Ethyl‐2‐methyl‐octane	ND	11.28 ± 2.12	85.15 ± 13.32	
66	Dodecane	ND	1.2 ± 0.34	ND	Alkane odor
67	Tetradecane	ND	0.45 ± 0.12	ND	Slightly sweet fragrance
68	Limonene	ND	ND	6.67 ± 3.75	Lemon and orange flavors
69	Maltol	1.80 ± 0.33	ND	ND	Sweet caramel flavor
70	2‐Pentylfuran	ND	55.84 ± 20.97	29.33 ± 3.26	Bean fragrance, fruit fragrance, vegetable fragrance
71	Octadecyl vinyl ether	ND	0.49 ± 0.17	ND	
72	Glycidyl isobutyl ether	ND	1.42 ± 0.46	2.22 ± 0.70	
	A total of 14	51.85	91.82	230.19	

*Note:* Data are expressed as the mean ± standard deviation. ND referred to no detection. QP, QY, and CY referred to quinoa porridge, quinoa yogurt, and commercial yogurt, respectively.

Compared QY with QP, it was found that fermentation significantly increased the types of flavor compounds, especially for other types (increased by 37.81%) and alcohols (increased by 90.03%), respectively. Compared with CY, the types and contents of volatile flavor in QY were more abundant, giving QY a unique flavor.

#### 3.2.5 The Total Phenol Content and Antioxidant Activity

**FIGURE 5:**
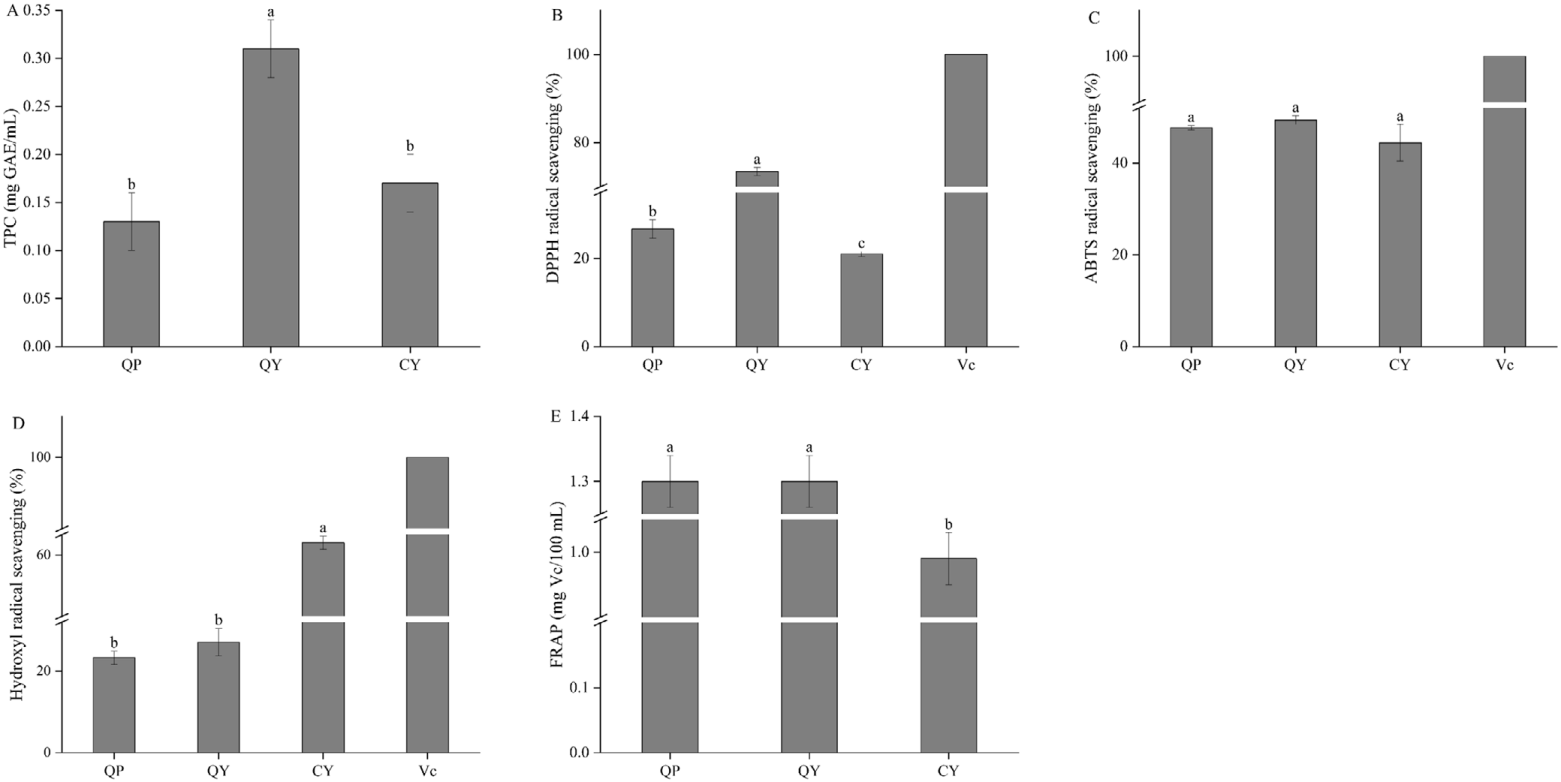
The TPC and antioxidant activity of QP, QY, and CY: (A) TPC; (B) DPPH radical scavenging; (C) ABTS radical scavenging; (D) Hydroxyl radical scavenging; (E) FRAP. Data are expressed as the mean ± standard deviation. Different lowercase letters indicate significant differences among the samples (*p* < 0.05). QP, QY, and CY refer to quinoa porridge, quinoa yogurt, and commercial yogurt, respectively.

The TPC determination results of QY were shown in Figure 5A. As can be seen from the figure, after the LAB fermentation, the TPC of QY was significantly higher than that of QP and CY (*p* < 0.05), which was consistent with the results of Chu et al. (2024). It was reported that quinoa was rich in phenolic compounds mainly existing in the bound form (Zannini et al. 2018). Fermentation could produce polyphenol oxidase, which helps to hydrolyze bound phenolic compounds and convert them into free forms (Lei et al. 2024); thereby the TPC has increased.

The antioxidant capacity of QY was reflected by DPPH, ABTS, hydroxyl radical, and FRAP, as shown in Figure 5B–E. The DPPH radical scavenging rate of QY was significantly higher than that of QP and CY (*p* < 0.05). It was found that the bound phenols were released under the hydrolyzation of enzymes produced by LAB, thereby increasing the TPC and antioxidant activity (Lei et al. 2024; Li et al. 2021). This result was consistent with the TPC result of this paper. In the present research, fermentation had no significant effect on the ABTS radical scavenging rate, hydroxyl radical scavenging rate, and FRAP (*p* > 0.05), which might be related to the detection method, changes in phenolic compounds and different mechanisms of action, or because the phenolic substances released combined with other molecules in the food matrix and were degraded by specific microbial enzyme‐active strains (Adebo et al. 2020).

### 3.3 Effects of Simulation of Gastrointestinal Digestion In Vitro on Antioxidant Capacity

**FIGURE 6:**
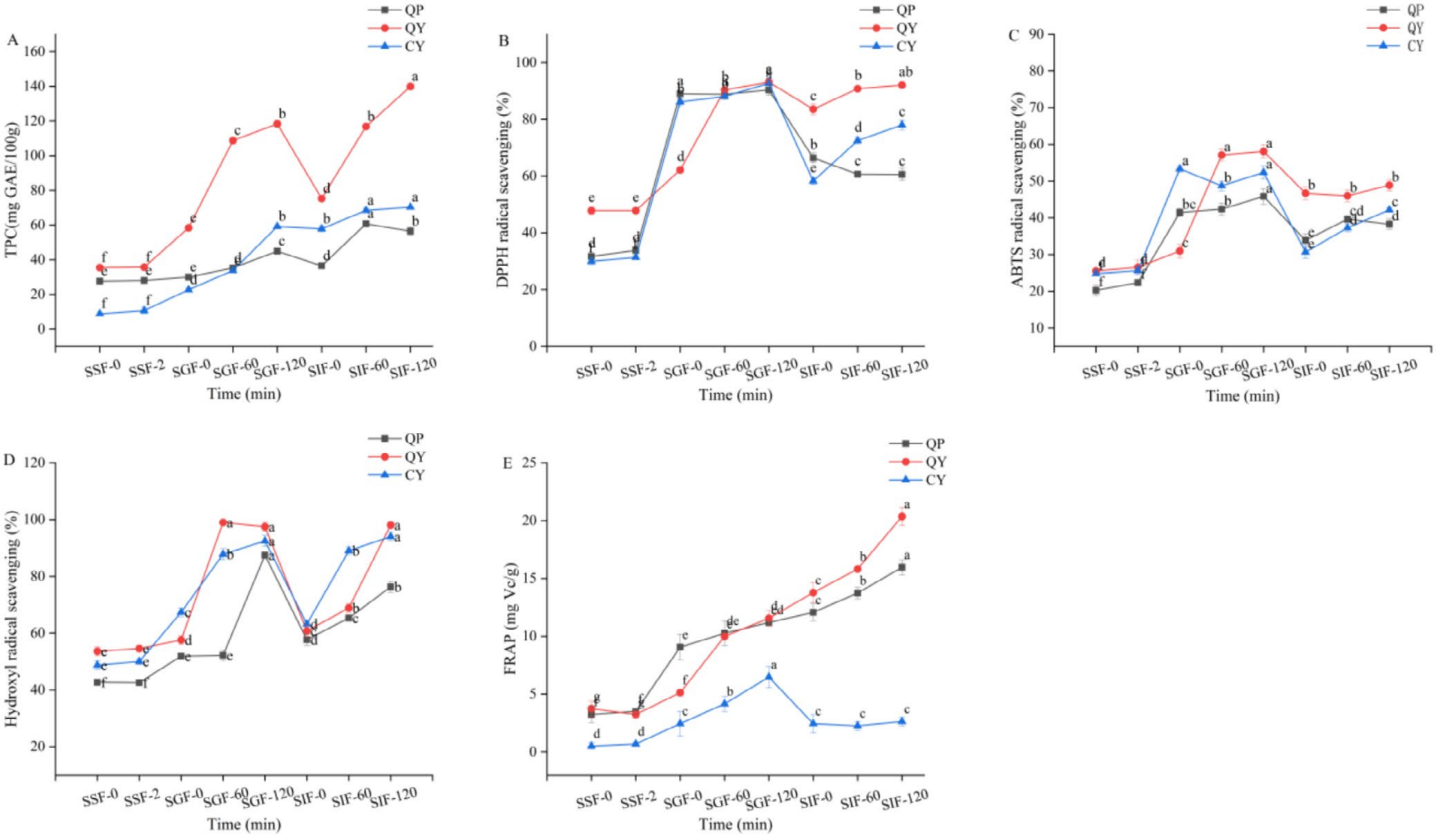
Changes of TPC and antioxidant activity during simulated in vitro gastrointestinal digestion: (A) TPC; (B) DPPH radical scavenging; (C) ABTS radical scavenging; (D) Hydroxyl radical scavenging; (E) FRAP. SSF‐0 and SSF‐2 indicated simulated saliva fluid digestion for 0 and 2 min, respectively. SGF‐0, SGF‐60, and SGF‐120 indicated simulated gastric fluid digestion for 0, 60, and 120 min, respectively. SIF‐0, SIF‐60, and SIF‐120 indicated simulated intestinal fluid digestion for 0, 60, and 120 min, respectively. Data are expressed as the mean ± standard deviation. Different lowercase letters within the same line indicate significant differences among the samples (*p* < 0.05). QP, QY, and CY referred to quinoa porridge, quinoa yogurt, and commercial yogurt, respectively.

The TPC, DPPH, ABTS, Hydroxyl radical, and FRAP after each digestion stage were determined in order to discover the influences of digestive juices. According to Figure 6, there was no significant change in these five indicators of QP, QY, and CY during the oral digestion stage (*p* > 0.05). Polyphenolic active substances in plants often exist in free, soluble conjugate, and insoluble bound forms; among them, free phenolic substances were unstable and easily degraded after gastrointestinal digestion (Sun et al. 2012). For QY, compared to the gastric digestion stage, the TPC and FRAP were significantly risen while ABTS was significantly reduced after the intestinal digestion stage (*p* < 0.05), but DPPH and Hydroxyl radical of QY had no significant change (*p* > 0.05). Unlike QY, the TPC was significantly risen whereas FRAP, DPPH, and ABTS radical were reduced (*p* < 0.05), while Hydroxyl radical of CY did not significantly change (*p* > 0.05). Although phenolic active substances were released to a certain extent during the intestinal digestion stage, the stability of TPC and other antioxidant active components in a weakly alkaline environment was poor in their free state; this resulted in the degradation of soluble polyphenolic substances, causing a loss of some of them (Liu et al. 2021). Moreover, the antioxidant capacity was the result of the joint action of many antioxidant substances in the raw materials, and changes in the environment from the stomach to the intestine may also lead to changes in the structure and interaction of total phenols and other antioxidant active components (Tong et al. 2024). In a word, after simulating the in vitro digestion process, the TPC and four antioxidant activities of QY were higher than those of QP and CY. In this paper, the trends of the results for four different antioxidant indicators were different. This may be related to the different determination methods, soluble proteins, small molecule peptides, free amino acids, vitamins, and other substances produced during the release of free phenolic substances in yogurt with an antioxidant role (Zielinski and Kozłowska 2000).

### 3.4 Storage Stability Evaluation

**FIGURE 7:**
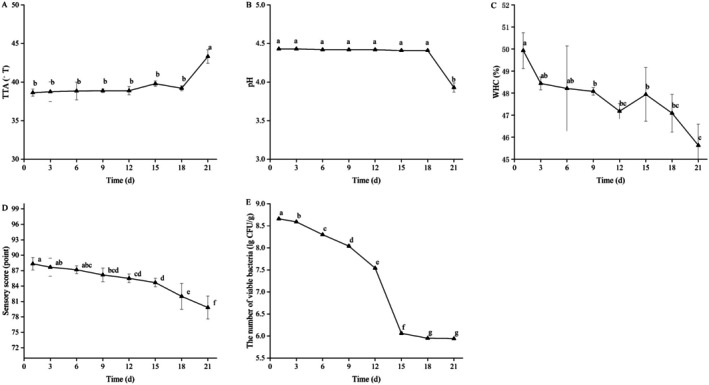
Changes in the physicochemical indicators of QY during storage: (A) TTA; (B) pH; (C) WHC; (D) Sensory score; (E) The number of viable bacteria. Data are expressed as the mean ± standard deviation. Different lowercase letters indicate significant differences in the sample over different storage periods.

During the 21‐day storage period, the TTA and pH of QY did not significantly change except for the last 3 days (Figure 7A~B). Although the life activities of LAB were inhibited under low‐temperature conditions, they still have relatively weak metabolic activities, and the acids continue to be produced during storage. This was often considered one of the reasons for yogurt spoilage (Anuyahong et al. 2020). The WHC showed a continuous downward trend during the storage period, but the decline was less than 5% (Figure 7C). It may be that yogurt was further fermented by LAB during storage, and the protein with a gel structure was degraded by the produced protease, leading to a decline in the stability of the gel network inside the yogurt (Lalou et al. 2017); the sensory scores have also decreased (Figure 7D). The number of LAB was one of the indicators to measure the stability of yogurt during storage. The LAB can regulate the human intestinal microbiota, promote intestinal peristalsis, and enhance the absorption of nutrients by the human body. Therefore, the more abundant the quantity, the more beneficial they are to the human body. According to Figure 7E, the number of viable bacteria (The *Streptococcus salivarius* subsp. *thermophilus* and *Lactobacillus delbrueckii* subsp. *bulgaricus*) in QY showed a steady decline during storage, meeting the national microbiological standards (The number of viable bacteria > 10^6^ CFU/g) within 15 days. Pathogenic bacteria such as *Escherichia coli* , *Salmonella*, and *Staphylococcus aureus* were not detected throughout the storage period, all meeting the national standards. Overall, the optimal storage period for yogurt was 18 days.

## 4 Conclusion

The results showed that the contents of protein, fat, total solids, reducing sugar, total sugar, essential amino acids, and total amino acids QY were 3.60% ± 0.01%, 2.08% ± 0.02%, 17.18% ± 0.63%, 14.73 ± 0.66 mg/g, 17.76 ± 1.17 mg/g, 5.61 ± 0.21 mg/g, and 15.56 ± 0.69 mg/g, respectively. The brightness of the QY significantly increased, with the red and yellow colors intensified, and the whiteness index reached 68.64 ± 1.57. In terms of texture, the hardness, adhesiveness, gumminess, and chewiness of QY significantly increased, resulting in a more compact three‐dimensional network‐like structure being formed. Furthermore, after fermentation, a series of organic acids were produced, such as lactic acid and succinic acid; additionally, 41 types of volatile flavor substances were generated, including esters, lactones, ketones, aldehydes, alcohols, acids, and other types. The in vitro simulated digestion significantly enhanced the TPC, DPPH, ABTS, FRAP, and hydroxyl radical scavenging ability. The optimal storage period for QY was found to be 18 days. This study provides new technical references and theoretical support for the development of other pure plant‐based functional yogurts. However, this article also has some shortcomings regarding other functional components present in QY and their health benefits. Therefore, in the next step, other biological activities of QY should be detected using multiple methods, and their functions, such as anti‐inflammatory effects and lipid‐lowering effects, should be further explored.

## Author Contributions

Qi Wang: conceptualization (lead), funding acquisition (lead). Qianyu Li: formal analysis (lead), data curation (lead), writing original draft (lead). Hong Zhang, Jingtong Wu: investigation (lead). Wenjun Qin: supervision (lead). Jin Cai: validation (lead).

## Ethics Statement

This study was approved by the Ethics Review Committee for Scientific Research at Shanxi University (Approval No. SXULL2025119).

## Consent

Written informed consent was obtained from all study participants.

## Conflicts of Interest

The authors declare no conflicts of interest.

## Data Availability

The data that support the findings of this study are available from the corresponding author upon reasonable request.
